# Tumour content ratio matters for detecting epidermal growth factor receptor mutation by cobas test in small biopsies; a retrospective study

**DOI:** 10.1186/s12885-020-6603-3

**Published:** 2020-02-06

**Authors:** Mariko Kogo, Daichi Fujimoto, Kazutaka Hosoya, Kazuma Nagata, Atsushi Nakagawa, Ryo Tachikawa, Daisuke Yamashita, Yuka Kitamura, Yukihiro Imai, Keisuke Tomii

**Affiliations:** 1 Department of Respiratory Medicine, Kobe City Medical Center General Hospital, 2-1-1 Minatojima-minamimachi, Chuo-ku, Kobe, Hyogo 650-0047 Japan; 2 Department of Pathology, Kobe City Medical Center General Hospital, 2-1-1 Minatojima-minamimachi, Chuo-ku, Kobe, Hyogo 650-0047 Japan; 3 Department of Pathology, Nagasaki University Graduate School of Biomedical Sciences, 1-12-4 Sakamoto, Nagasaki, 852-8523 Japan

**Keywords:** *EGFR* mutation, Cobas, PCR clamp, Non-small cell lung cancer, Osimertinib

## Abstract

**Background:**

Recent studies indicate the benefit of treatment with osimertinib over that with conventional epidermal growth factor receptor (EGFR) tyrosine kinase inhibitors (TKI) for untreated *EGFR*-mutated non-small cell lung cancer (NSCLC). Cobas ver2 is the only companion diagnostic method for detecting *EGFR* mutations with osimertinib treatment. We clinically experience false negative cases with this test, but its actual sensitivity is unknown. Moreover, no study has suggested the importance of tumour dissection, and most facilities do not routinely perform them on small biopsies. The purpose of this study was to evaluate the sensitivity of cobas in clinical practice and clarify the role of dissection as a component of the cobas testing.

**Methods:**

We examined 132 patients with *EGFR*-mutated NSCLC diagnosed by bronchoscopy and confirmed with PCR clamp. Patients were tested with cobas and the *EGFR*-positive rate was calculated. Samples with undetected *EGFR* mutations were retested after tumour dissection and the rate of samples whose *EGFR* mutation was corrected to positive was assessed. To evaluate tumour cellularity, the tumour content ratio was assessed by calculating tumour cell count over the total cell count on the slide.

**Results:**

The positive rate of *EGFR* mutation identification was 76% with cobas, although *EGFR* mutation-negative patients retained responses to TKI therapy equivalent to positive patients did; however, the tumour content ratio of negative samples was significantly lower than that of positive samples. Twenty-nine negative samples underwent dissection and 24% were corrected to positive. Moreover, 53% of the samples with a tumour content ratio below 10% was negative for cobas, but 33% of these turned positive after dissection.

**Conclusions:**

Cobas had a high false negative rate in clinical practice, and tumour content ratio might be associated with this rate. Dissection could improve the sensitivity of cobas, especially in samples with low tumour cellularity.

## Background

Epidermal growth factor receptor (EGFR) tyrosine kinase inhibitors (TKIs) exhibit clinically significant therapeutic responses in patients with non-small cell lung cancer (NSCLC) that harbour *EGFR* driver mutations [1–4]. Recently, osimertinib has been shown to exert remarkable effects against untreated *EGFR* mutation-positive advanced NSCLC as well as those with EGFR-TKI-sensitizing and *EGFR* T790 M resistance mutations [4]. This third generation TKIs can bind irreversibly to the EGFR kinase by targeting the cystine-797 residue in the ATP binding site via covalent bond formation and a phase 3 trial revealed prolonged progression-free survival with a similar safety profile and lower rates of serious adverse events compared to standard EGFR-TKIs [5]. Based on these results, osimertinib is now a key drug for the first line treatment for patients with *EGFR*-mutated NSCLC.

Cobas ver2 companion tissue diagnostic test is the only companion diagnostic test for the detection of *EGFR* mutations when introducing osimertinib. It detects *EGFR* mutations of more than 5% against 95% wild-type alleles. Although its sensitivity is considered equivalent to other conventional methods, we clinically experience many false negative cases with the cobas test that are positive with other tests [6]. To confirm good sensitivity, the manufacturer of the cobas test encourages tumour dissection of the specimen for maintaining high tumour cellularity. However, no study has clarified the efficacy of this dissection and not a few facilities are incapable of routinely performing this dissection in the real world.

In this study, we evaluated the actual sensitivity of the cobas test with small biopsies performed as a usual clinical practice to identify the importance of tumour cellularity in the specimen slice when identifying *EGFR* mutations. We also evaluated the efficacy of sample dissection on a slide for improving the sensitivity of this testing method.

## Methods

### Study design and population

For this study, we included eligible patients diagnosed with NSCLC by a bronchoscopic biopsy at our hospital between January 2007 to January 2017 and confirmed that they expressed *EGFR* mutations by an improved version of PCR clamp [7]. In October 2013, the improved version of the PCR clamp (which can detect S768I and L833X mutations, Ex 20 insersion in addition to the *EGFR* mutations detectable with previous version) was introduced in our hospital, and therefore, we retested samples obtained before October 2013 with the improved version and included those that tested positive. The following clinicopathological factors were obtained from medical charts and analysed: age, sex, detected *EGFR* mutation, smoking status, stage [8], 1st line EGFR-TKI therapy and the best response to the therapy according to the Response Evaluation Criteria in Solid Tumours (version 1.1), and progression free survival (PFS) calculated from the initiation of the TKI therapy until the date of disease progression.

### Detection of *EGFR* mutations and analysis of cobas test sensitivity

We evaluated the sensitivity of the cobas method for *EGFR* mutation detection, using the PCR clamp results as a control. Formalin-fixed, paraffin-embedded tumour samples from the first bronchoscopy biopsy were cut at 4 μm and deparaffinised. Samples for improved PCR clamp retesting were collected at the same time as the samples used for the cobas test as required. As suggested by the guidelines [9], we selected the samples with as minimal necrosis as possible, as we routinely do for the genomic tests in the clinical practice. One blinded pathologist reviewed a serial haematoxylin and eosin-stained section from each sample and calculated the tumour content ratio (i.e. the ratio of tumour cells over total cells) on the slide.

### Tumour dissection on a slide using microscopy

Samples that had *EGFR* mutations confirmed by PCR clamp, but negative by cobas, underwent dissection at a 4-μm slice that was mounted on a slide. Tumour tissues were manually dissected with a scalpel under a microscope using a serial haematoxylin and eosin-stained section as a guide. After dissection, *EGFR* status was re-tested with the cobas method and the rate of samples now found to be positive for *EGFR* mutation was assessed.

### Statistical analysis

The categories of detected *EGFR* mutation and the response to TKI therapy and tumour content ratio were compared with chi-square test and with Wilcoxon test, respectively, between *EGFR*-positive and negative patients with cobas. The Kaplan-Meier method and log-rank test were used to evaluate the PFS. The ability of the tumour content ratio of the sample to predict *EGFR* mutation detection with cobas was determined using a receiver operating characteristic curve (ROC) analysis. A two-tailed *p*-value of < 0.05 indicated statistical significance and all analyses were performed using JMP 11 software (SAS Institute, Cary, NC, USA).

## Results

### Patient characteristics

**Fig. 1:**
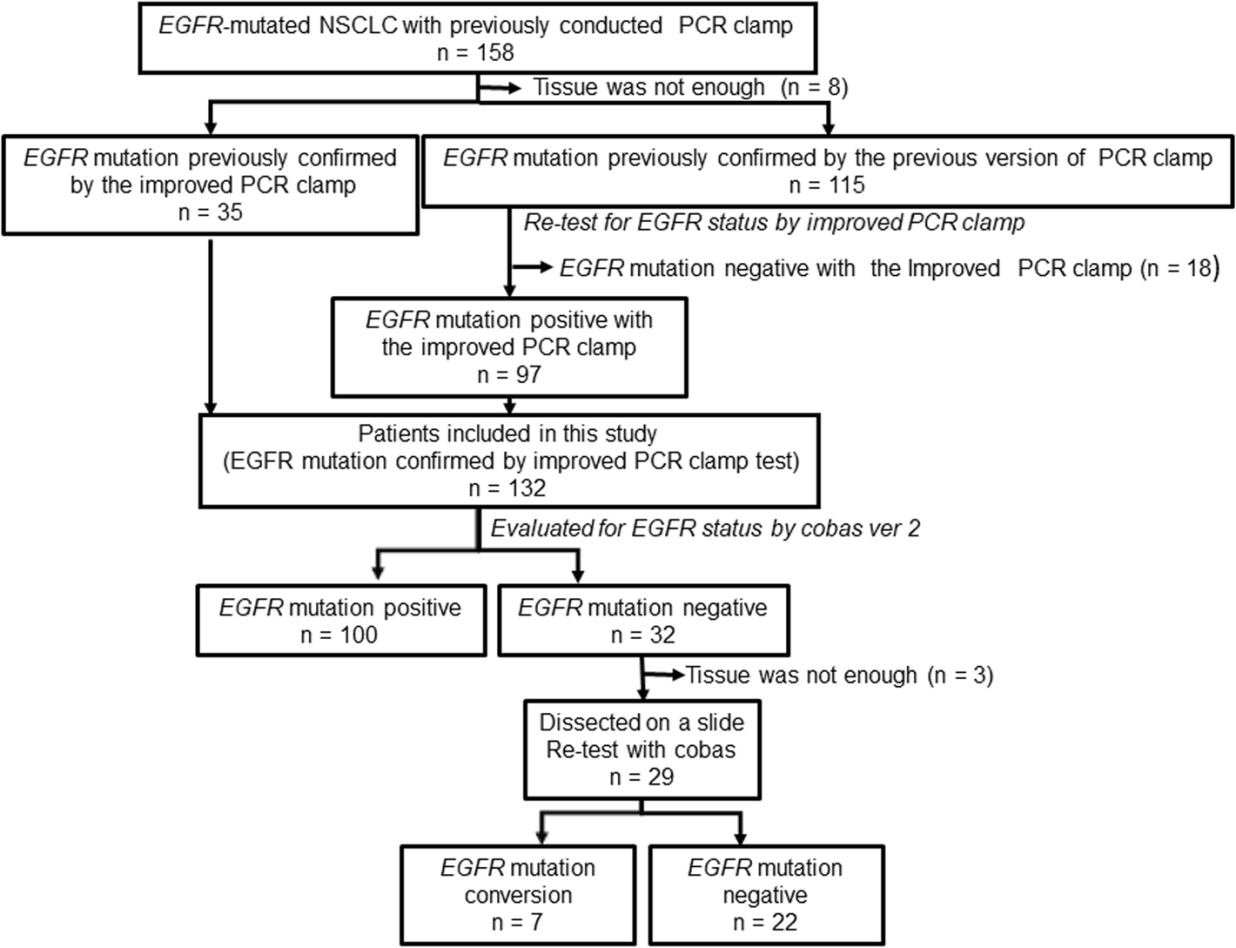
Patient Flowchart. We examined 132 patients with *EGFR*-mutated NSCLC confirmed by PCR clamp. The positive rate of EGFR mutation identification with cobas was 76%. Twenty-nine EGFR mutation negative samples underwent dissection and 24% were corrected to positive. Abbreviations: EGFR; epidermal growth factor receptor, NSCLC; non-small cell lung cancer

During the study period, 158 patients were diagnosed with *EGFR*-mutated NSCLC by bronchoscopy (Fig. 1). Eight patients were excluded due to inadequate amount of tissue for our study. One hundred and fifteen patients required retesting for *EGFR* status with the improved PCR clamp method and 97 patients was proven to contain *EGFR* mutations. The 18 patients who showed negative results for *EGFR* mutation following retesting with the improved PCR clamp were excluded from the study. Finally, 132 patients (35 patients with *EGFR* mutations previously confirmed by the improved PCR clamp and 97 patients with *EGFR* mutations confirmed by the improved PCR clamp test at the initiation of this study) were included and underwent cobas testing. Patient characteristics are shown in Table 1. The median patient age was 69 years (interquartile range [IQR] 61–76). Also 128 (97%) were diagnosed with adenocarcinoma, 3 (2%) with squamous cell carcinoma and 1 (1%) with NSCLC containing morphological squamous cell and adenocarcinoma patterns. The majority of *EGFR* mutations detected by PCR clamp were a 19 deletion (49%) and an exon 21 L858R substitution (43%). One hundred and ten patients received EGFR-TKI therapy, 23 of which showed negative results with the cobas method, with a 73% response rate and median PFS of 12.2 months (95% CI 9.7–13.7). Histopathologically, 126 out of the 132 samples (95%) contained tumour cell counts of more than 100. The median of the tumour content ratio calculated on the slide was 17% with an IQR of 10–31%.

**Table 1:** Patient characteristics

	All patients *n* = 132
Age, year, median (IQR)	69 (61–76)
Male, n (%)	49 (37)
*EGFR* mutation with PCR clamp, n (%)	
19del	65 (49)
L858R	57 (43)
L861Q	4 (3)
G719X	5 (5)
T790 M	1 (1)
Smoking Status, n (%)	
Never	84 (64)
Former/Current	48 (36)
Tumour histological type, n (%)	
Adenocarcinoma	128 (97)
Squamous cell carcinoma	3 (2)
NSCLC with morphological squamous cell and adenocarcinoma patterns	1 (1)
Stage, n (%)	
1	19 (14)
2	8 (6)
3A	8 (6)
3B	7 (5)
4	90 (68)
TKI therapy, n (%)	110 (83)
Gefitinib^a^	64 (58)
Erlotinib^a^	23 (21)
Afatinib^a^	22 (20)
Naquotinib^a^	1 (1)
Number of chemotherapies before TKI therapy, n (%) ^a^	
0 regimen	65 (59)
1 regimen	37 (34)
2 regimens	6 (5)
3 regimens	2 (2)
Response to TKI, n (%)^a^	
PR/CR	81 (73)
SD	12 (11)
PD	11 (10)
NA	6 (5)
PFS for the first TKI therapy, m, median (95%CI)	12.2 (9.7–13.7)
Tumour content ratio^b^, %, median (IQR)	17 (10–31)

*Abbreviations*: *IQR* interquartile range, *EGFR* epidermal growth factor receptor, *NSCLC* non-small cell carcinoma, *TKI* tyrosine kinase inhibitors, *PR* partial response, *CR* Complete Response, *SD* Stable Disease, *PD* Progressive Disease, *NA* not available, *PFS* progression free survival

^a^Analysis of 110 patients who received TKI therapy. 23 of the patients showed negative results with the cobas method

^b^Tumour cell count over the total cell count conducted on a slide-mounted biopsy sample

### Cobas test sensitivity

Among the 132 samples tested, 100 (76%) were positive and 32 (24%) were negative for *EGFR* mutations with the cobas test (Fig. 1). In the subgroup analysis of 97 patients whose samples for both PCR clamp and cobas were prepared simultaneously at the beginning of this study, we found 26% had false negative result with cobas. Among the patients that had detectable *EGFR* mutations with the cobas method, we also observed consistent *EGFR* mutation profiles using PCR clamp except for one who carried both L858R and T790 M mutations identified by PCR clamp, but only the L858R mutation was identified with the cobas test (Table 2). No significant difference was observed in the TKI response rate (74% vs 65%, *p* = 0.59) or the PFS for the first TKI therapy (median 12.2 months [95%CI 9.3–16] vs 12.4 months [95%CI 7.3–13.3], *p* = 0.24) between *EGFR* positive and negative samples with cobas. The *EGFR* positive samples had a significantly higher tumour content ratio than negative samples did (median 19% (11–32) vs 11% (5–18), *p* = 0.002), indicating that the tumour cellularity might contribute to the false negative results of *EGFR* mutation identification with the cobas testing.

**Table 2:** Comparison of the characteristics of patients with positive and negative *EGFR* mutation cobas test results

	*EGFR* positive *n* = 100	*EGFR* negative *n* = 32	*p* value
*EGFR* mutation with PCR clamp, n (%)			0.012
19del	55 (55)	10 (31)	
L858R	36 (36)	21 (66)	
L861Q	4 (4)	0 (0)	
G719X	5 (5)	1 (3)	
T790 M	1 (1)^a^	0 (0)	
Tumour histological type, n (%)			0.52
Adenocarcinoma	96 (96)	32 (100)	
Squamous cell carcinoma	3 (3)	0 (0)	
NSCLC with morphological squamous cell and adenocarcinoma patterns	1 (1)	0 (0)	
TKI therapy, n (%)	87 (87)	23 (72)	0.29
Gefitinib^a^	52 (60)	12 (52)	
Erlotinib^a^	16 (18)	7 (30)	
Afatinib^a^	18 (21)	4 (17)	
Naquotinib^a^	1 (1)	0 (0)	
Number of chemotherapies before TKI therapy, n (%)^a^			.08
0 regimen	56 (64)	9 (39)	
1 regimen	27 (31)	10 (43)	
2 regimens	3 (3)	3 (13)	
3 regimens	1 (1)	1 (4)	
Response to TKI, n (%)^b^			0.59
PR/CR	64 (74)	15 (65)	
SD	8 (9)	4 (17)	
PD	8 (9)	3 (13)	
NA	7 (8)	1 (4)	
PFS for the first TKI therapy, m, median (95%CI)	12.2 (9.3–16)	12.4 (7.3–13.3)	0.24
Tumour content ratio^c^, %, median (IQR)	19 (11–32)	11 (5–18)	0.002

*Abbreviations*: *EGFR* epidermal growth factor receptor, *NSCLC* non-small cell carcinoma, *TKI* tyrosine kinase inhibitors, *PR* partial response, *CR* Complete Response, *SD* Stable Disease, *PD* Progressive Disease, *NA* not available, *PFS* progression free survival, *IQR* interquartile range

^a^Double positive with L858R and T790 M by PCR clamp test and only L858R by cobas test

^b^Analysis of 110 patients who received TKI therapy. 23 of the patients showed negative results with the cobas method

^c^Tumour cell count over the total cell count conducted on a slide-mounted biopsy sample

### Effect of dissection of sample-mounted slides on *EGFR* detection with cobas

Twenty nine out of 32 samples negative for *EGFR* mutation by the cobas test were manually dissected on a slide under a microscope, of which seven (24%) were found to be positive for *EGFR* mutation (Fig. 1). The final false negative rate for the cobas method among all 132 patients was calculated to be 17% (22 / 132) after dissection. Three of the patients who changed from negative to positive *EGFR* mutation after dissection were able to be evaluated for a response to TKI therapy and all achieved response with PFS that was comparable to *EGFR* positive patients tested with cobas without dissection (13.1 months, 13.1 months and 5.7 months, respectively).

### Tumour content ratio and cobas sensitivity

**Fig. 2:**
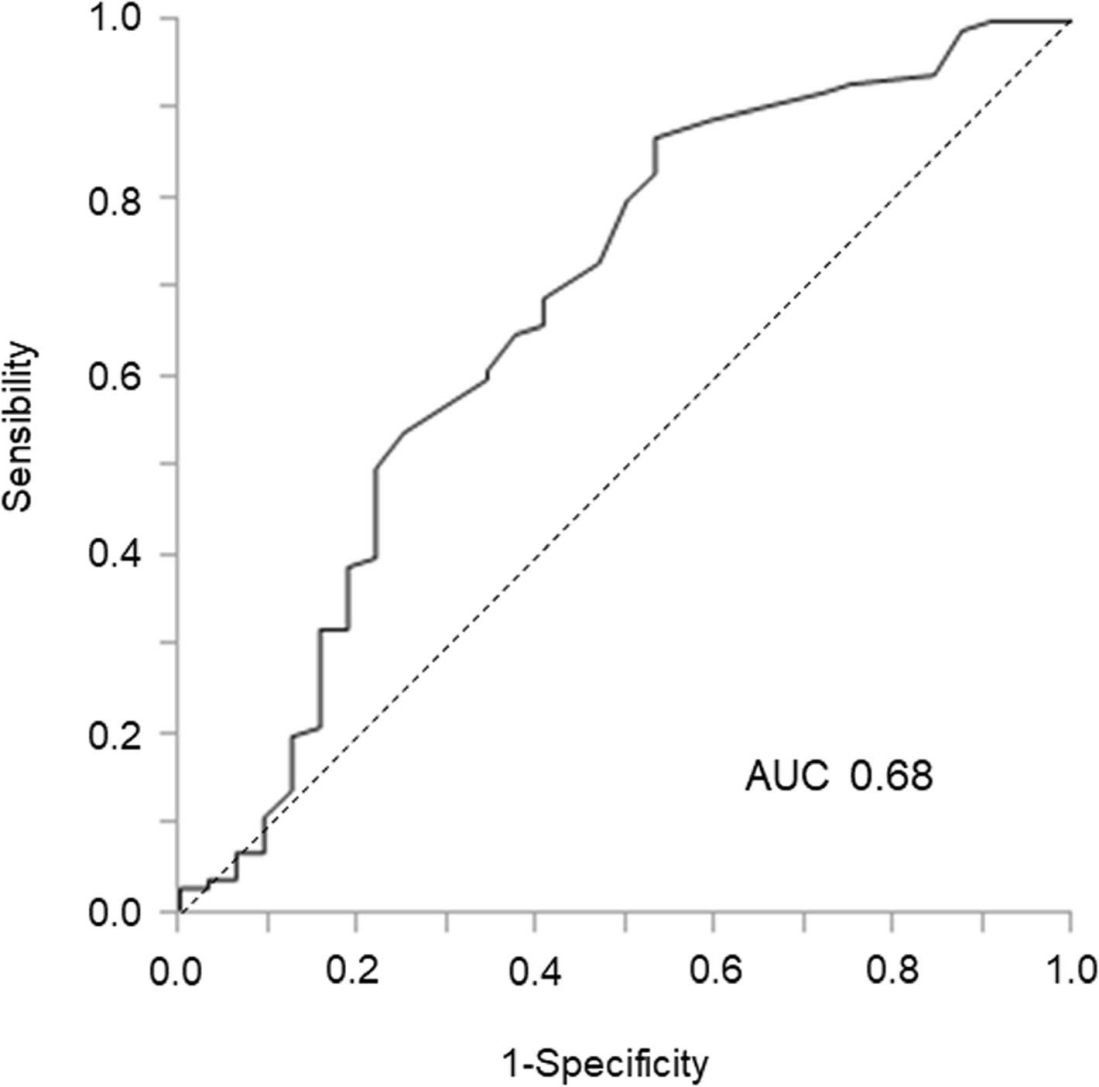
Receiver operating characteristic curve analysis of tumour content ratios to predict EGFR detection by cobas. The area under the curve (AUC) was 0.68 with a cut-off reference value of 9% (sensitivity 0.87, specificity 0.47)

ROC analysis of the tumour content ratio from a biopsy-mounted slide was used to predict *EGFR* identification confirmed by cobas and showed the area under the curve (AUC) was 0.68 with a cut-off reference value of 9% (sensitivity 0.87, specificity 0.47) (Fig. 2). The *EGFR* detection rate was 53 and 83% among samples with a tumour content ratio < 10% and ≥ 10%, respectively (Table 3). After dissection, 33% of the samples that were negative for *EGFR* mutation with a tumour content ratio < 10% were corrected to *EGFR* positive, although only 14% recovery was observed in the samples with negative *EGFR* samples with a tumour content more than 10%.

**Table 3:** Tumour content ratio and cobas sensitivity

	Tumour content ratio < 10%	Tumour content ratio ≥ 10%
*EGFR* positive samples with cobas/total samples, n (%)	17/32 (53)	83/100 (83)
*EGFR* positive samples after dissection/total number of dissected samples, n (%)	5/15 (33)	2/14^a^ (14)

*Abbreviations*: *EGFR* epidermal growth factor receptor

^a^Three of the *EGFR* negative samples were not included due to an inadequate amount of tissue for cobas retesting

## Discussion

This is one of the first studies that aims to evaluate the sensitivity of the cobas test in a clinical setting and investigates the role of microscopic dissection of small biopsies to overcome its false negative results. This study revealed that the *EGFR* mutation detection failure of cobas occurred in about 25% of *EGFR* mutated patients in a clinical setting. Moreover, we demonstrated that the false negative rate of cobas went up to 50% when the tumour content ratio was below 10% and that dissection on a slide could improve the test sensitivity.

Now that EGFR-TKI therapy dramatically improved the prognosis for *EGFR* mutated NSCLC [4, 10–12], the detection of such mutations is a critical step when managing lung cancer patients. Recent evidence has shown the superiority of osimeritinib over first- and second-generation EGFR-TKIs and is an approved first line treatment for patients with *EGFR*-mutated NSCLC [4]. Cobas ver2 is the only osimertinib companion diagnostic tool to identify *EGFR* mutations. Although it is considered to be as precise as conventional methods [13], there are only a few studies that compare the sensitivity of these methods [14]. One study compared the detection abilities of *EGFR* mutations between cobas and PCR clamp with 15 re-biopsied samples and reported consistent detection accuracy between the two assays except for one sample that tested positive with PCR clamp but negative with cobas [14]. In our study, we found a 25% false negative rate with cobas compared with that of PCR clamp. This lower sensitivity in cobas was consistent with that revealed in the report [14], and, moreover, our study successfully calculated the high false negative rate of cobas with a large population.

This low positive rate of cobas was not reported at the correlation tests of *EGFR* mutation identification performed previously to the approval of the cobas method [15]. We hypothesized that the different experimental conditions in our study for clinical setting and in laboratory research for sensitivity test could be one of the important factors of this false negative results. As we analysed tumour samples taken for usual clinical practice, our study included samples with low tumour content ratio. With the trait of cobas, these samples with limited tumour cellularity potentially cause *EGFR* mutation detection failure. The cobas method is an in vitro diagnostic tool that applies an allele-specific PCR method. Matching failure of primers at annealing tends to occur in samples with a lower rate of *EGFR* mutations over total *EGFR* and this determines the sensitivity of the cobas method for *EGFR* mutation identification. Furthermore, the PCR clamp, we used as a control for *EGFR* mutation identification, is a major laboratory developed test with the sensitivity for *EGFR* mutations as high as 1%. This high sensitivity of PCR clamp was confirmed by masking wild type *EGFR* with specific nucleic acids during the PCR process [11]. However, as PCR clamp was independently developed in a major laboratory centre, the problem of uniformisation between laboratories should be considered, while the consistency of the cobas method is guaranteed as an in vitro diagnostic. These specific processes in PCR and non-commercial optimisation of PCR clamp could affect both high sensitivity and false positive rates in *EGFR* mutation identification, especially when the response to EGFR-TKI therapy is considered. In our cohort, *EGFR* mutation was determined by PCR clamp at inclusion, and patients had an equivalent EGFR-TKI response to previous studies [10, 11]. Moreover, both cobas-positive and -negative patients showed no difference in the response. From these results, we concluded that our result revealed the high false negative rate of the cobas method instead of high false positive rate of the PCR clamp.

Our study also suggests the association between a low tumour content ratio and high false negative rates of *EGFR* identification in a small biopsy cohort in the cobas method. Although AUC of the ROC curve could only demonstrate a weak relationship, importantly, when the tumour content ratio was below 10%, the false negative rate was as high as 50%. Therefore, we focused on the microscopic tumour dissection on a slide to confirm high tumour cellularity of the samples and improve its low sensitivity. Indeed, the manufacturer of cobas encourages tumour dissection. However, the dissection is not routinely performed in clinical settings, especially on small biopsies. This might be due to a cost-performance effect or the limitation from pathologist manpower at the hospital, but we have to acknowledge that the evidence of dissection in conjunction with cobas is lacking. Here, we revealed that 24% of the cobas negative samples showed positive *EGFR* mutations after performing tumour dissection on a slide with bronchoscopic biopsied samples. Especially in a subgroup with tumour content ratio below 10%, one-third of the *EGFR* mutation false negative samples with the cobas method at first turned to be positive at the re-test after the dissection. This study provides some of the first evidence for the importance of maintaining high tumour content ratio when testing for *EGFR* mutations and encourages microscopic tumour dissection with small biopsies.

Our study did have some technical limitation that were important to consider during our analysis and interpretation. Firstly, we could only confirm *EGFR* mutation with PCR clamp and cobas performed at the same time in 97 samples out of the 132 included patients. With the rest 35 samples, PCR clamp was conducted prior to the test with cobas method at the beginning of this study. In these samples, the best part of tissue samples for the genomic testing were already taken at the time of the PCR clamp testing, and samples with worse quality could have been used for the cobas testing as compared to those used for the initial PCR clamp tests, especially the cobas testing after the tumour dissection that was conducted later in our study. Although this could somewhat contribute to the high false negative rate in the cobas tests, the false negative rate was consistent even in samples where the sample slides for cobas method were made at the same time as those collected for PCR clamp at the beginning of our study. We also demonstrated the recovery of *EGFR* mutation detection after the dissection for samples whose slides were made after the cobas test showed a negative result. Based on these results, we conclude that the false negative rate of cobas observed in our study reliably represents the clinical setting and that time of sampling does not significantly alter the different diagnostic outcomes.

Secondly, we observed that 16% of the samples retested with an improved PCR clamp at the beginning of the study were negative for *EGFR* mutation. The long retention period from the time the samples were collected to the time of the *EGFR* mutation test may have contributed to this result. Indeed, the Japanese Society of Pathology reports that DNA deteriorates in old samples, showing a decreased Q-value of DNA over time [9]. The varying sample collection times for testing *EGFR* mutations may, therefore, also be another factor contributing to observed negative result. However, our cohort showed consistent characteristics including response to the TKIs, with those of a previous study [10, 11], suggesting the validity of our study results for representing the sensitivity of cobas and the utility of the dissection in clinical settings.

Other limitation is that the number of the false negative results with cobas was so small that the association between the tumour content ratio and cobas sensitivity was not well described. Further, the precise histology including patterns of invasive adenocarcinoma were not available preventing us from investigating the association between tumour histology and *EGFR* mutation detection with cobas. Moreover, this is a retrospective analysis with samples collected more than 10 years earlier. There is also a possibility of confounding factors we could not evaluate and further studies would be needed to address these challenges. However, we emphasize the importance of this study as one of the first to point out the high false negative rate in the realistic use of the cobas method and importance of dissection even in a small sample for maintaining its sensitivity.

## Conclusion

Our data revealed that high false negative rate of cobas was as high as 50% when the tumour content ratio was below 10% in small biopsy specimens. Biopsy dissection on a slide could be one strategy for improving the false negative results during patient tumour evaluation.

## Data Availability

The datasets used and/or analysed during the current study are available from the corresponding author on reasonable request.

## Abbreviations

AUC: Area under the curve
EGFR: Epidermal growth factor receptor
IQR: Interquartile range
NSCLC: Non-small cell lung cancer
PFS: Progression free survival
ROC: Receiver operating characteristic curve
TKI: Tyrosine kinase inhibitors
